# Serum Pepsinogen Values and *Helicobacter pylori* Status among Control Subjects of a Nested Case-Control Study in the JACC study

**DOI:** 10.2188/jea.15.S126

**Published:** 2005-08-18

**Authors:** Shogo Kikuchi, Kiyoko Yagyu, Yuki Obata, Lin Yingsong, Hiroshi Yatsuya, Yoshiharu Hoshiyama, Takaaki Kondo, Kiyoshi Sakata, Tetsuya Mizoue, Noritaka Tokui, Yoshihisa Fujino, Akiko Tamakoshi, Hideaki Toyoshima, Teruo Ishibashi, Norihiko Hayakawa, Takesumi Yoshimura

**Affiliations:** 1 Department of Public Health, Aichi Medical University School of Medicine.; 2 Department of Public Health/Health Information Dynamics, Nagoya University Graduate School of Medicine.; 3 Department of Public Health, Showa University School of Medicine.; 4 Department of Medical Technology, Nagoya University School of Health Sciences.; 5 Department of Public Health, Wakayama Medical University.; 6 Department of Preventive Medicine, Graduate School of Medical Sciences, Kyushu University.; 7 Department of Clinical Epidemiology, Institute of Industrial Ecological Science, University of Occupational and Environmental Health.; 8 Fukuoka Institute of Occupational Health.; 9 Department of Preventive Medicine/Biostatistics and Medical Decision Making, Nagoya University Graduate School of Medicine.; 10 Asama General Hospital.; 11 Department of Epidemiology, Research Institute for Radiation Biology and Medicine, Hiroshima University.; 12 Fukuoka Institute of Health and Environmental Science.

**Keywords:** Helicobacter pylori, Pepsinogen A, Pepsinogen B, Seroepidemiologic Studies, Japan

## Abstract

BACKGROUD: *Helicobacter pylori* infection and serum pepsinogen values are strongly related with stomach cancer. The aim of this study was to know what were these factors among general population.

METHODS: Subjects were randomly selected 633 control subjects in a nested case-control study for risk of stomach cancer. Most of them were from rural areas of Japan. Using frozen sera, pepsinogen I (PG I) and II (PG II) values and *H. pylori* antibody were measured. Those with PG I less than 50 ng/mL and the ratio of PG I to PG II (PG I/II) was less than 2.0 were defined as severe, those with PG I less than 70 ng/ml and PG I/II less than 3.0 were defined as mild and the other subjects were defined as no serological atrophy.

RESULTS: About 70% of the subjects were *H. pylori* seropositive and the seroprevalence did no depend on age or sex. Percentages of those with severe serological atrophy increased with age from 10% in those aged 40-49 years to 38% in 70 and more, and percentages of those with mild serological atrophy were about 30% independent of age.

CONCLUSIONS: The subjects, who were expected to represent populations of rural area of Japan, had high prevalence of both *H. pylori* infection and serological atrophy of gastric mucosa. These facts should be considered in discussing results of the nested case-control study.

*Helicobacter pylori* infection is one of the causes of stomach cancer. Many epidemiologic studies so far have argued relationship between *H. pylori* and risk of stomach cancer.^1^^,^^2^ However, several negative results have been obtained even in East Asian countries, where both mortality of stomach cancer and prevalence of *H. pylori* are high.^3^ One explanation for the negative results is very high prevalence of *H. pylori* in the general population or control subjects.^3^ Another explanation is spontaneous eradication of *H. pylori* and consequent seroreversion between the time of diagnosis and when *H. pylori* plays a role in gastric carcinogenesis.^4^ Serum pepsinogen is a marker of both inflammation and atrophy of gastric mucosa.^5^ It is also related to the risk of stomach cancer, and either a low value of pepsinogen I (PG I),^6^^,^^7^ a high value of pepsinogen II (PG II), or a low value of PG I to PG II ratio indicates an increased risk of stomach cancer.^8^

The Japan Collaborative Cohort Study (JACC Study) for Evaluation of Cancer Risk sponsored by the Ministry of Education, Science, Sports and Culture of Japan (Monbusho) is a cohort study initiated between 1988 and 1990 when apparently healthy residents aged 40-79 years were enrolled as a basic cohort population from 45 areas throughout Japan.^9^ The subjects answered a questionnaire including lifestyle factors and past histories as baseline information of the study. A peripheral blood sample was collected from some of the subjects. A total of 39,242 subjects provided their sera when they attended general health check programs. The main aim of the JACC Study is to investigate risk factors of cancers. A nested case-control study was conducted in the JACC Study to evaluate the relationships between serum markers and risk of stomach cancer, and serum pepsinogen and *H. pylori* antibody was measured using sera from 164 death and 161 incidence cases of stomach cancer and 635 control subjects.^10^^,^^11^

The current study was conducted to know what was the *H. pylori* and serum pepsinogen status, what factors were related to status of the serum markers among the control subjects, and what was the effect of them on the case-control study.

## METHODS

Subjects were selected from those who provided their sera for the JACC Study in 1988-1990.^9^ They were 635 control subjects who were matched for 164 death and 161 incidence cases of stomach cancer in the JACC Study. Matched for age (±6 year), sex and study area with each case subject, the control subjects were randomly selected from those who were alive without diagnosis or history of stomach cancer at the time of death or diagnosis of the case subject. Two subjects, whose pepsinogen values were not measured because of lack in sera, were excluded from the study and 633 subjects were enrolled.

Serum PG I, PG II values, and *H. pylori* antibody were measured using the sera from the cases and control subjects. Sera were separated from the blood samples at laboratories in each study area and stored at -80°C until analysis. All the samples were assayed in 1999 by trained staff at a single laboratory who were blinded to the case/control status of the individuals. Serum levels of *H. pylori* antibody and PG I and PG II were measured using JHM-CAP (Kyowa Medex Co. Ltd., Tokyo) and RIAbeads Pepsinogen I and II kits (Dainabot Co. Ltd., Tokyo), respectively. *H. pylori* status was defined according to the manufacturer’s instructions. When the titer of *H. pylori* antibody was less than 2.3, it was defined as negative, and defined as positive when not less than 2.3. Using serum pepsinogen values, the subjects were classified into three levels of inflammation/atrophy of gastric mucosa (expressed as serological atrophy below): severe, mild and no. Those with PG I less than 50 ng/mL and the ratio of PG I to PG II (PG I/II) was less than 2.0 were defined as severe, those with PG I less than 70 ng/mL and PG I/II less than 3.0 were defined as mild and the other subjects were defined as no serological atrophy. The subjects were divided into 6 groups by levels of gastric mucosal serological atrophy and *H. pylor*i status: severe/positive, severe/negative, mild/positive, mild/negative, no/positive and no/negative. Then age (40-49, 50-59, 60-69, 70-79 years) and sex distribution of *H. pylori* infection and serological atrophy determined by serum pepsinogen values was analyzed. In order to confirm difference between rural and urban area, prevalence of *H. pylori* infection and serological atrophy was compared between the subjects from a city with a population of a million people and the other subjects.

Relationships between *H. pylori* infection or severe serological atrophy and factors in the baseline questionnaire were evaluated using logistic regression models with adjustment for age and sex. The evaluated factors were father’s and/or mother’s history of stomach cancer, history of mass-survey for stomach cancer, pregnancy, intake of salted foods, dried fish, drinking and smoking habits.

## RESULTS

**Figure 1.**
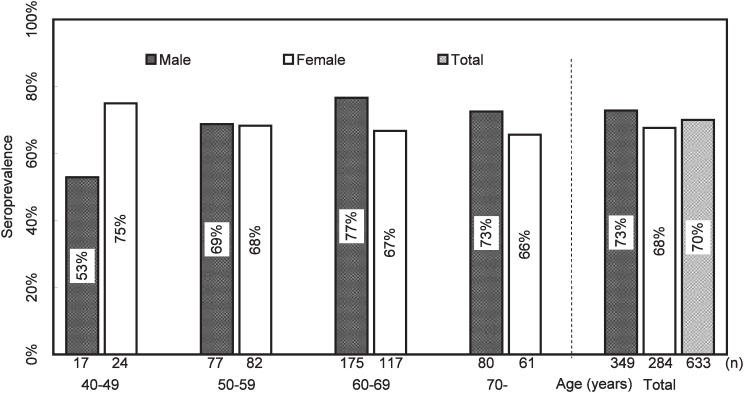
*Helicobacter pylori* seroprevalence with respect to age and sex.

**Figure 2.**
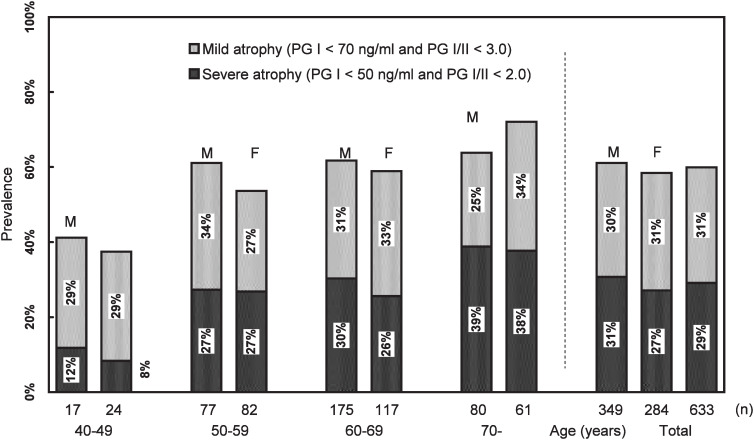
Serum pepsinogen status with respect to age and sex.

Age and sex distribution and *H. pylori* serology of the subjects are shown in Figure 1. *H. pylori* prevalence did not depend on age or sex. About 70% of the subjects were *H. pylori* seropositive. Male subjects in their 40s showed exceptional 53% of seroprevalence. Percentages of those with severe serological atrophy increased with age from about 10% in those aged 40-49 years to about 38% in 70 and more in both male and female subjects (Figure 2). Percentages of those with mild serological atrophy were about 30% and did not depend on age or sex. In those aged 40-49 years, percentages of those without serological atrophy was about 60%, whereas that was less than 50% in those over 50 years of age.

**Figure 3.**
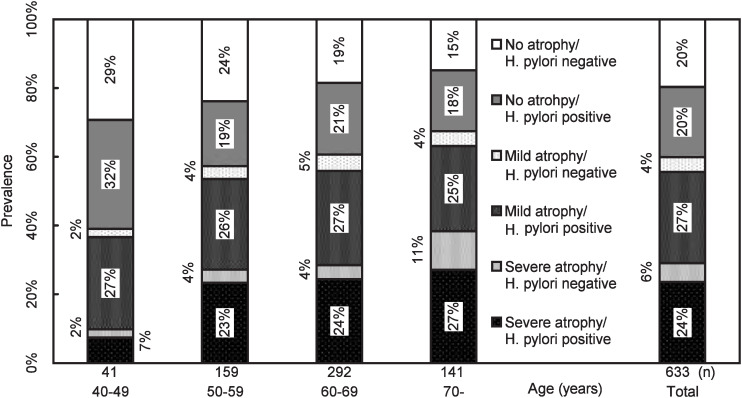
Disribution of serum pepsinogen and *Helicobacter pylori* status.

Figure 3 shows age distribution of the 6 groups determined by serological atrophy and *H. pylori* status. As similar results were obtained between both sexs of subjects, only total results are shown. Percentages of the no/negative decreased with age from 30% to 15%, while those of the severe/positive increased from 7% to 27%. Percentages of the mild/negative and the mild/positive groups did not change over age. The severe/negative increased with age and were frequent among those with severe serological atrophy in those aged 70 years.

Subjects from a large city showed lower seroprevalence of *H. pylori* (54%) than those from the other areas (72%), but no difference was observed in frequency of serological atrophy (Table 1).

**Table 1.** Comparison of H.pylori and serological atrophy prevalence between a large city and other areas.

Area	n*	*H. pylori* positive (%)	Serological atrophy (%)		

			No	Mild	Severe
A large city	37	54	43	30	27
Others	596	72	40	31	29
		p=0.039			p=0.920

*: number of subjects.

No factors were related with *H. pylori* status (Table 2). On the other hand, history of mass survey for stomach cancer and increased intake of salted foods compared with when young were positively, and intake of dried fish were negatively related with severe serological atrophy.

**Table 2.** Related factors with Helicobacter pylori serology and low pepsinogen.

Factor	Category	*Helicobacter pylori*	p-value*	Severe inflammation/atrophy^†^	p-value*
Father’s history of stomach cancer	No	375 / 532 (71%)^‡^	0.558	154 / 532 (28%)	0.087
	Yes	27 / 36 (75%)		15 / 36 (42%)	

Mother’s history of stomach cancer	No	389 / 548 (71%)	0.511	165 / 548 (30%)	0.498
	Yes	14 / 22 (64%)		5 / 22 (23%)	

Father’s and/or Mother’s history of stomach cancer	No	342 / 485 (71%)	0.987	142 / 485 (29%)	0.268
	Yes	38 / 54 (70%)		20 / 54 (37%)	

History of mass-survey for stomach cancer	No	197 / 286 (69%)	0.843	69 / 286 (24%)	0.018
	Yes	157 / 220 (71%)		75 / 220 (34%)	

Salted foods	Very fond of	7 / 10 (70%)	0.954	5 / 10 (50%)	0.403
	The other	361 / 513 (70%)		148 / 513 (29%)	

More salted foods than when young	No	358 / 513 (70%)	0.337	148 / 513 (29%)	0.011
	Yes	6 / 7 (86%)		5 / 7 (71%)	

Intake of dired fish (grilled)	Less than 3 times a week	272 / 387 (70%)	0.698	122 / 387 (32%)	0.038
	More frequent	121 / 166 (73%)		40 / 166 (24%)	

Drinking habit	Current drinker	196 / 286 (69%)	0.798	81 / 286 (28%)	0.706
	Ex-drinker	12 / 15 (80%)		4 / 15 (27%)	
	Never-drinker	217 / 301 (72%)		92 / 301 (31%)	

Smoking habit	Current smoker	210 / 311 (68%)	0.156	89 / 311 (29%)	0.571
	Ex-smoker	84 / 107 (79%)		36 / 107 (34%)	
	Never-smoker	122 / 166 (74%)		46 / 166 (28%)	

Pregnancy	0-2 times	47 / 64 (73%)	0.464	12 / 64 (19%)	0.171
	3 and more	130 / 191 (68%)		55 / 191 (29%)	

* : Adjusted for age and gender using logistic regression.

† : PG I < 50 ng/mL and PG I/II < 2.0

‡ : Positive / subjects (%)

## DISCUSSION

This cross sectional serological study has shown prevalence of *H. pylori*, mild and severe serological atrophy among control subjects of a nested case-control study, who were not diagnosed as stomach cancer until death or diagnosis of their matched case subjects.

Our previous study, collecting sera from workers in the Tokyo Metropolitan Area, Japan in 1989, reported that seroprevalence was 40% in those aged 39-44 years, 49% in 45-54, 56% in 55-64 and 56 % in 65 and older.^12^ Even though sera were collected almost concurrently, there were differences in seroprevalence between this study and our previous study whose subjects were from urban area. Several explanations seem to be possible for the difference. In this study, *H. pylori* antibody was measured using a domestic kit, which has shown better accuracy than imported kits.^13^^,^^14^ Our previous study used an imported kit “Pilika-Plate G Helicobacter” which is the same kit as “IgG-GAP.” However, it seems to be impossible to explain the difference in seroprevalence only by the used kits, because differences in sensitivity and specificity between the two kinds of kits are at most 10% and 6%, respectively.^13^^,^^14^ Another explanation is that prevalence of *H. pylori* is different between in rural areas and urban areas.^15^ Actually, the subjects from a large city with a population of a million people showed similar seroprevalence to our previous study. As most of the subjects were from rural area, seroprevalence in the JACC Study may be high compared with seroprevalence in 1988-90 in urban areas including our previous study.

Many studies have reported that seroprevalence increases with age, which is different from this study.^12^^,^^16^ The increase of seroprevalence with age is due to two reasons: decreasing trend of *H. pylori* prevalence that is provoked by improvement in sanitary conditions and accumulation of continuing infection.^17^ In another study of ours, seroconversion rate was lower than seroreversion rate among subjects over 40 years of age,^18^^,^^19^ which means accumulation of continuing infection affects little on the increase of seroprevalence with age. In a Japanese study showing increase of *H. pylori* seroprevalence with age, seroprevalence did not increase with age among those who were born before 1950.^20^ The subjects of the current study were born before 1950, and infection of *H. pylori* is frequent under 5 years of age.^21^ Improvement in social overhead capital including sanitary conditions was little at least in rural areas of Japan before 1955,^22^ which may be one of the reasons for the *H. pylori* prevalence independent of age in the studies.

Prevalence of severe serological atrophy increased with age, whereas mild serological atrophy did not depend on age. Although this study is a cross-sectional one, migration of subjects from no to mild and from mild to severe serological atrophy with time may be suggested. When *H. pylori* serology is considered together with serological atrophy, those, who were *H. pylori* seronegative and had severe serological atrophy, increased with age and were about 11% among those over 70 years of age. As gastric mucosal atrophy is rare without *H. pylori* infection^23^ and seroreversion is not a seldom phenomenon among Japanese subjects,^18^ it is expected that many of these subjects had history of *H. pylori* infection and *H. pylori* had disappeared.

Studies to date have reported that family history of stomach cancer,^24^ smoking and drinking habits^12^ are associated with *H. pylori* serology. However, in this study *H. pylori* status was not related with the family history or lifestyle factors. The study reporting negative association of smoking and drinking habits with *H. pylori* seropositivity recruited more than 8000 subjects.^12^ Although the results were insignificant, current smokers and current drinkers showed lower seroprevalence in this study. The power of the study may be one of the reasons for the different results. The different results on family history of stomach cancer may be because higher *H. pylori* seroprevalence in this study. The high seroprevalence may have concealed or diluted the association.

Severe serological atrophy was positively associated with history of mass-survey for stomach cancer. One possible explanation for the association is that those with severe serological atrophy tended to have a symptom in the stomach, which may have taken them to the mass-survey program. Although this association is important to improve effectiveness of mass-survey programs for stomach cancer, sample size of this study is too small to do a conclusive discussion. Well-designed studies are needed on this point.

Increase of salted foods intake was positively and intake of grilled dried fish was negatively associated with severe serological atrophy. Intake of both salted foods and grilled dried fish is known to elevate risk for stomach cancer.^25^^,^^26^ It seems to be difficult to explain the inverse association of these factors. Only possible explanation is as follows. A part of those with severe gastric mucosal atrophy suffered some discomfort from intake of grilled dried fish, which did not accelerate atrophy. On the other hand, the seven subjects who answered that intake of salted foods increased than when young really had highly salted frequently, and atrophy of gastric mucosa was accelerated in their stomach. They did not feel any discomfort from intake of highly salted foods even though they have severe gastric mucosal atrophy. However, the latter relationship may be unstable compared with the former one because the number of subjects who increased salt intake was too small.

The subjects of the current study are control subjects of a nested case-control study who were not diagnosed as stomach cancer during the follow-up period. They were born before 1950 and were randomly selected from almost all study areas of the cohort study. Although there might be a bias that the subjects of the current study are more interested in their health conditions than the other residents of the areas because they gave their sera mainly at the time of general health check programs, they are expected to represent the population of their resident areas. It is expected that the population of the study areas, who were born before 1950, have high prevalence of both *H. pylori* infection and serological atrophy of gastric mucosa. These facts should be considered in discussing results of the nested case-control study using the subjects.

## MEMBER LIST OF THE JACC STUDY GROUP

The present investigators involved, with the co-authorship of this paper, in the JACC Study and their affiliations are as follows: Dr. Akiko Tamakoshi (present chairman of the study group), Nagoya University Graduate School of Medicine; Dr. Mitsuru Mori, Sapporo Medical University School of Medicine; Dr. Yutaka Motohashi, Akita University School of Medicine; Dr. Ichiro Tsuji, Tohoku University Graduate School of Medicine; Dr. Yosikazu Nakamura, Jichi Medical School; Dr. Hiroyasu Iso, Institute of Community Medicine, University of Tsukuba; Dr. Haruo Mikami, Chiba Cancer Center; Dr. Yutaka Inaba, Juntendo University School of Medicine; Dr. Yoshiharu Hoshiyama, University of Human Arts and Sciences; Dr. Hiroshi Suzuki, Niigata University School of Medicine; Dr. Hiroyuki Shimizu, Gifu University School of Medicine; Dr. Hideaki Toyoshima, Nagoya University Graduate School of Medicine; Dr. Kenji Wakai, Aichi Cancer Center Research Institute; Dr. Shinkan Tokudome, Nagoya City University Graduate School of Medical Sciences; Dr. Yoshinori Ito, Fujita Health University School of Health Sciences; Dr. Shuji Hashimoto, Fujita Health University School of Medicine; Dr. Shogo Kikuchi, Aichi Medical University School of Medicine; Dr. Akio Koizumi, Graduate School of Medicine and Faculty of Medicine, Kyoto University; Dr. Takashi Kawamura, Kyoto University Center for Student Health; Dr. Yoshiyuki Watanabe, Kyoto Prefectural University of Medicine Graduate School of Medical Science; Dr. Tsuneharu Miki, Graduate School of Medical Science, Kyoto Prefectural University of Medicine; Dr. Chigusa Date, Faculty of Human Environmental Sciences, Mukogawa Women’s University ; Dr. Kiyomi Sakata, Wakayama Medical University; Dr. Takayuki Nose, Tottori University Faculty of Medicine; Dr. Norihiko Hayakawa, Research Institute for Radiation Biology and Medicine, Hiroshima University; Dr. Takesumi Yoshimura, Fukuoka Institute of Health and Environmental Sciences; Dr. Akira Shibata, Kurume University School of Medicine; Dr. Naoyuki Okamoto, Kanagawa Cancer Center; Dr. Hideo Shio, Moriyama Municipal Hospital; Dr. Yoshiyuki Ohno, Asahi Rosai Hospital; Dr. Tomoyuki Kitagawa, Cancer Institute of the Japanese Foundation for Cancer Research; Dr. Toshio Kuroki, Gifu University; and Dr. Kazuo Tajima, Aichi Cancer Center Research Institute.
